# The Length of *SNCA* Rep1 Microsatellite May Influence Cognitive Evolution in Parkinson’s Disease

**DOI:** 10.3389/fneur.2018.00213

**Published:** 2018-03-29

**Authors:** Lucia Corrado, Fabiola De Marchi, Sara Tunesi, Gaia Donata Oggioni, Miryam Carecchio, Luca Magistrelli, Silvana Tesei, Giulio Riboldazzi, Alessio Di Fonzo, Clarissa Locci, Ilaria Trezzi, Roberta Zangaglia, Cristina Cereda, Sandra D’Alfonso, Corrado Magnani, Giacomo P. Comi, Giorgio Bono, Claudio Pacchetti, Roberto Cantello, Stefano Goldwurm, Cristoforo Comi

**Affiliations:** ^1^Laboratory of Genetics, Department of Health Sciences, University of Piemonte Orientale, Novara, Italy; ^2^Section of Neurology, Department of Translational Medicine, University of Piemonte Orientale, Novara, Italy; ^3^Unit of Medical Statistics and Cancer Epidemiology, Department of Translational Medicine, University of Piemonte Orientale, Novara, Italy; ^4^Center for Cancer Epidemiology and Prevention (CPO), University Hospital “Città della Salute e della Scienza di Torino”, Turin, Italy; ^5^Parkinson’s Disease and Movement Disorders Center, Ospedale di Circolo Fondazione Macchi, University of Insubria, Varese, Italy; ^6^Parkinson Institute, ASST Gaetano Pini-CTO (Formerly ICP), Milan, Italy; ^7^Neuroscience Section, Department of Pathophysiology and Transplantation, IRCCS Foundation Ca’ Granda Ospedale Maggiore Policlinico, Dino Ferrari Center, University of Milan, Milan, Italy; ^8^Parkinson’s Disease and Movement Disorders Unit, C. Mondino National Institute of Neurology Foundation, IRCCS, Pavia, Italy; ^9^Genomic and Post-Genomic Center, C. Mondino National Institute of Neurology Foundation, IRCCS, Pavia, Italy

**Keywords:** dementia, hallucinations, genetic markers, disease progression, Parkinson’s disease

## Abstract

**Background:**

Alpha-synuclein is a constituent of Lewy bodies and mutations of its gene cause familial Parkinson’s disease (PD). A previous study showed that a variant of the alpha-synuclein gene (*SNCA*), namely the 263 bp allele of Rep1 was associated with faster motor progression in PD. On the contrary, a recent report failed to detect a detrimental effect of Rep1 263 on both motor and cognitive outcomes in PD. Aim of this study was to evaluate the influence of the Rep1 variants on disease progression in PD patients.

**Methods:**

We recruited and genotyped for *SNCA* Rep1 426 PD patients with age at onset ≥40 years and disease duration ≥4 years. We then analyzed frequency and time of occurrence of wearing-off, dyskinesia, freezing of gait, visual hallucinations, and dementia using a multivariate Cox’s proportional hazards regression model.

**Results:**

*SNCA* Rep1 263 carriers showed significantly increased risk of both dementia (HR = 3.03) and visual hallucinations (HR = 2.69) compared to 263 non-carriers. Risk of motor complications did not differ in the two groups.

**Conclusion:**

*SNCA* Rep1 263 allele is associated with a worse cognitive outcome in PD.

## Introduction

Parkinson’s disease (PD) is the second most frequent neurodegenerative disease (1), and is clinically characterized by the presence of asymmetric motor signs, including resting tremor, rigidity, and bradykinesia (2). Nonetheless, non-motor symptoms, such as mood deflection, anosmia, and sleep disturbances may also be present and can at times pre-date motor impairment (3). As disease progresses, further disabling symptoms appear, such as posture and gait impairment on the motor side, and cognitive decline and hallucinations in the non-motor domain (4, 5).

Parkinson’s disease neuropathology is characterized by the loss of dopaminergic neurons and the presence of Lewy bodies (LBs) in surviving neurons (6, 7). LBs contain fibrils composed of alpha-synuclein, a small protein involved in synaptic vesicle trafficking and neurotransmitter release (7), but also in more widespread functions, including the triggering of neuroinflammatory processes (8). Duplication and triplication of alpha-synuclein gene (*SNCA*) cause dominant early-onset PD, suggesting that overexpression of wild-type alpha-synuclein is sufficient to cause the disease (9). The amount of alpha-synuclein is relevant in sporadic disease as well, since its expression is higher in brain tissue from PD patients compared to control tissue (10). Furthermore, genome-wide association studies revealed that *SNCA* variations are associated with sporadic PD development (11).

Variations in the complex microsatellite D4S3481 (known as Rep-1), located approximately 10 kb upstream of the translational start of *SNCA*, have been reported to increase PD risk. *SNCA* Rep1 is essentially triallelic (259, 261, and 263 base pairs in length) and a meta-analysis of association studies showed higher frequency of 263 bp allele in cases compared to controls (12). Furthermore, the 259 bp allele was found to be associated with a decreased risk of PD, being more frequent in controls than in cases, whereas no relevant effect was observed for the 261 bp allele (12). To date, data on the role of *SNCA* common variants in PD progression are conflicting. Ritz et al. analyzed 232 PD patients and found that the risk of faster motor decline, assessed by UPDRS, was fourfold increased in carriers of the Rep1 263 bp promoter variant (13). Thereafter, a study in which outcome was measured in terms of life expectancy, did not detect any association between *SNCA* Rep1 genotypes and risk of death in 6,154 PD cases (14). More recently, Markopoulou et al. (15) analyzed the correlation between *SNCA* Rep1 genotypes and both motor and cognitive outcomes, assessed by telephone interview, in a large cohort of PD patients. Surprisingly, they found an opposite role of the microsatellite variants, with shorter alleles providing worse outcomes.

On this background, the aim of our study was to investigate the effect of *SNCA* Rep1 on disease progression in a cohort of Italian PD patients, clinically characterized through the collection of solid and reliable milestones of disease evolution.

## Patients and Methods

### Patients

426 patients (249 males) with PD (2) were enrolled according to the following inclusion criteria: age at onset ≥40 years, longitudinal follow-up ≥ 4 years. All patients were of Italian origin and were enrolled at the following Movement Disorders Centers: (1) University of Piemonte Orientale, Novara, (2) University of Insubria, Varese, (3) IRCCS Foundation Ca’ Granda Ospedale Maggiore Policlinico, Dino Ferrari Center, Neuroscience Section, University of Milan, (4) C. Mondino National Institute of Neurology Foundation, IRCCS, Pavia, (5) Parkinson Institute, ASST Gaetano Pini-CTO, Milan. The Parkinson Institute cohort (144 patients) had been previously analyzed in both a case-control study assessing the role of *SNCA* variants in PD susceptibility (16) and in the collaborative GEO-PD study on survival (14).

Patients who had at least one first- or second degree relative with a diagnosis of primary parkinsonism, and/or age at onset ≤50 years and/or peculiar clinical features had been previously analyzed to exclude pathogenic mutations of known PD-related genes (*SNCA, LRRK2, Parkin, PINK1, and DJ-1*) according to EFNS guidelines (17).

This study was approved by the Ethics Committee of the University of Piemonte Orientale (identification code 65/16). Patients were included in the study after having read and signed an informed consent form for research purpose.

### Assessment of PD Progression

All recruited patients had been longitudinally evaluated every 6 months at each center by a team of neurologist’s expert in movement disorders. The mean follow-up duration was 11.3 years. Clinical records were retrospectively analyzed taking into consideration only variables that had been assessed in each center. Agreement was found on the following parameters: gender, age at onset, disease duration, and family history. Furthermore, the presence and time of onset of the following motor and non-motor complications was recorded: wearing-off, dyskinesia, freezing of gait, visual hallucinations, and dementia. Dementia was diagnosed with a comprehensive cognitive evaluation according to MDS criteria (18).

### Determination of *SNCA* Variants

Genomic DNA was extracted from peripheral blood using standard procedures. We analyzed the microsatellite *SNCA* Rep1 in the entire cohort (426 subjects). The *SNCA* Rep1 region was amplified through PCR from genomic DNA using the primer pair: 5′-GACTGGCCCAAGATTAACCA-3′ (fluorescently labeled with 6-FAM) and 5′-CCTGGCATATTTGATTGCAA-3′. PCR products were resolved by capillary electrophoresis on an ABI-3130XL DNA Analyzer (Applied Biosystem, Foster City, CA, USA) using GeenScan-500 ROX (Applied Biosystem) as molecular weight marker. Allelic sizes were assessed using the GeneMapper 4.0 software.

To confirm the accuracy of the genotyping method, we sequenced several individuals representative of each genotype as standard samples.

### Statistical Analysis

Allelic distribution was assessed for Hardy–Weinberg equilibrium (HWE) with Fisher’s exact test.

*SNCA* Rep1 was analyzed using a dominant genetic model (e.g., subjects 263 carriers vs 263 non-carriers) as previously described (13).

Continuous variables were reported as median and 25th and 75th percentile, categorical variables were presented as frequencies (counts) and percentages. Allelic frequencies and family history were compared with the χ^2^ test, differences between age at onset and disease duration were assessed with Mann–Whitney *U* test.

The influence of the *SNCA* variant carrier status on disease natural history was investigated performing time-to-event analysis: wearing-off, dyskinesia, freezing of gait, visual hallucinations, and dementia were considered as separate outcomes. Every patient contributed time of observation from disease onset to complication under study or the last assessment. Individual analyses were performed for each complication under study and cumulative incidences were estimated using the Kaplan–Meier (KM) methods. Log-rank test was used to examine univariate association with outcome. Multivariate Cox’s proportional hazards (PHs) regression model was fitted to obtain the hazard ratio (HR) for *SNCA* variant carrier status adjusted by gender, age at PD onset, and recruitment center.

Proportional hazard model was assessed by regression scaled Schoenfeld residuals against the log time. All *p*-values are two-tailed and the significance cut-off was *p* < 0.05. Statistical analysis was performed using STATA v14.

## Results

Demographic and clinical features of the 426 PD patients are summarized in Table 1.

**Table 1:** Demographic and clinical features of study population.

	*n* = 426
Male, *n*, (%)	249 (58.5%)
Age at onset, median (25–75^th^)	62 (55–68)
Age at assessment, median (25–75^th^)	74.0 (68–79)
Disease duration, median (25–75^th^)	11.0 (8–14)
Family history, *n*, (%)	81 (19.0%)
Wearing-off, *n*, (%)	231 (54.2%)
Dyskinesia, *n*, (%)	196 (46.0%)
Freezing of gait, *n*, (%)	169 (39.7%)
Visual hallucinations, *n*, (%)	85 (20.0%)
Dementia, *n*, (%)	77 (18.1%)

Observed frequencies of *SNCA* Rep1 genotypes were in HWE (*p* = 0.19) (Table S1 in Supplementary Material).

Disease duration and family history did not differ significantly between Rep1 263 carriers (*n* = 44) and 263 non-carriers (*n* = 382) (*p* = 0.088, Mann–Whitney *U* test, and *p* = 0.797, χ^2^ test). On the contrary, the former group showed significantly earlier age at onset compared to the latter (*p* = 0.016, Mann–Whitney *U* test) (Table S2 in Supplementary Material).

**Figure 1:**
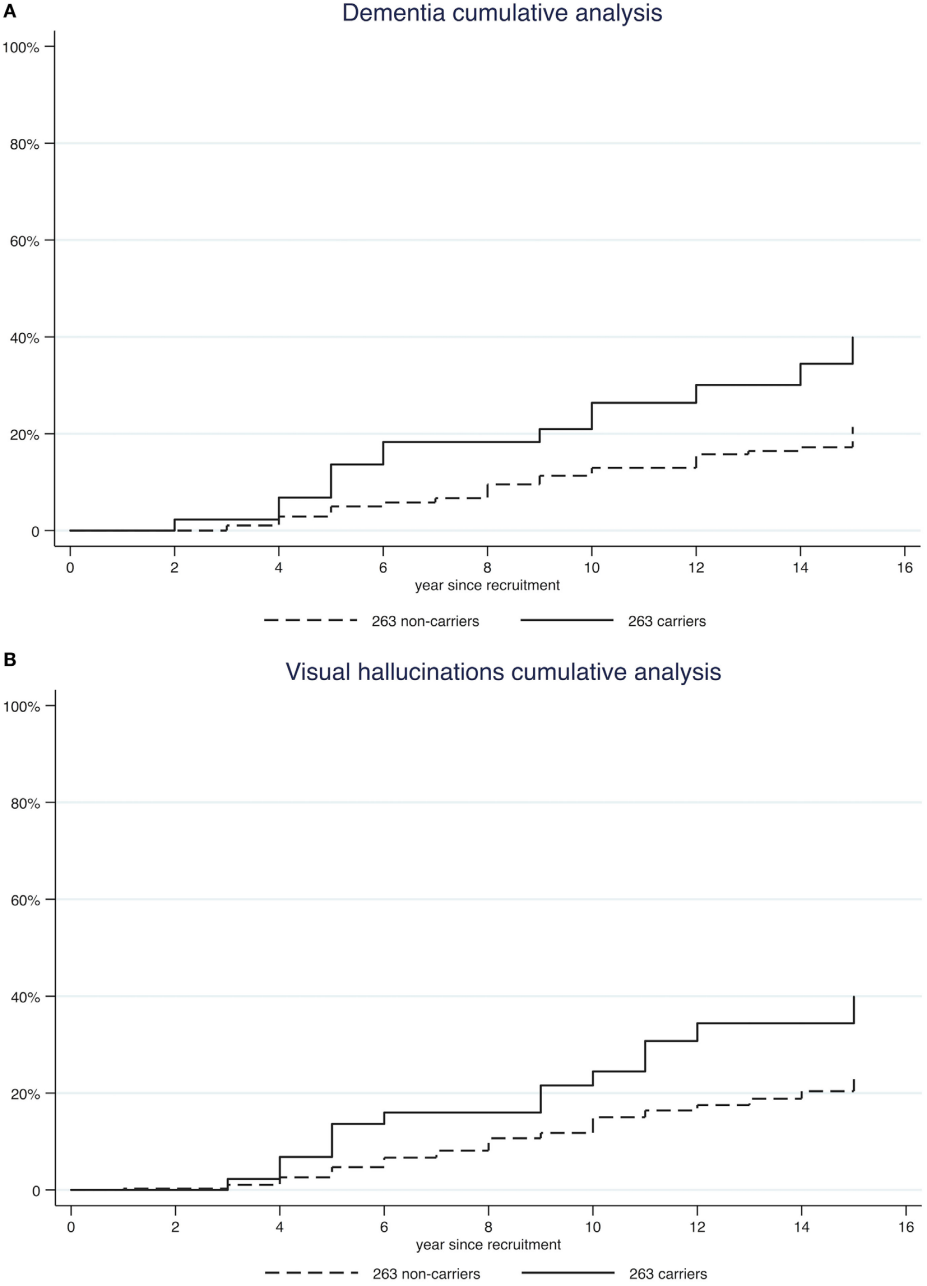

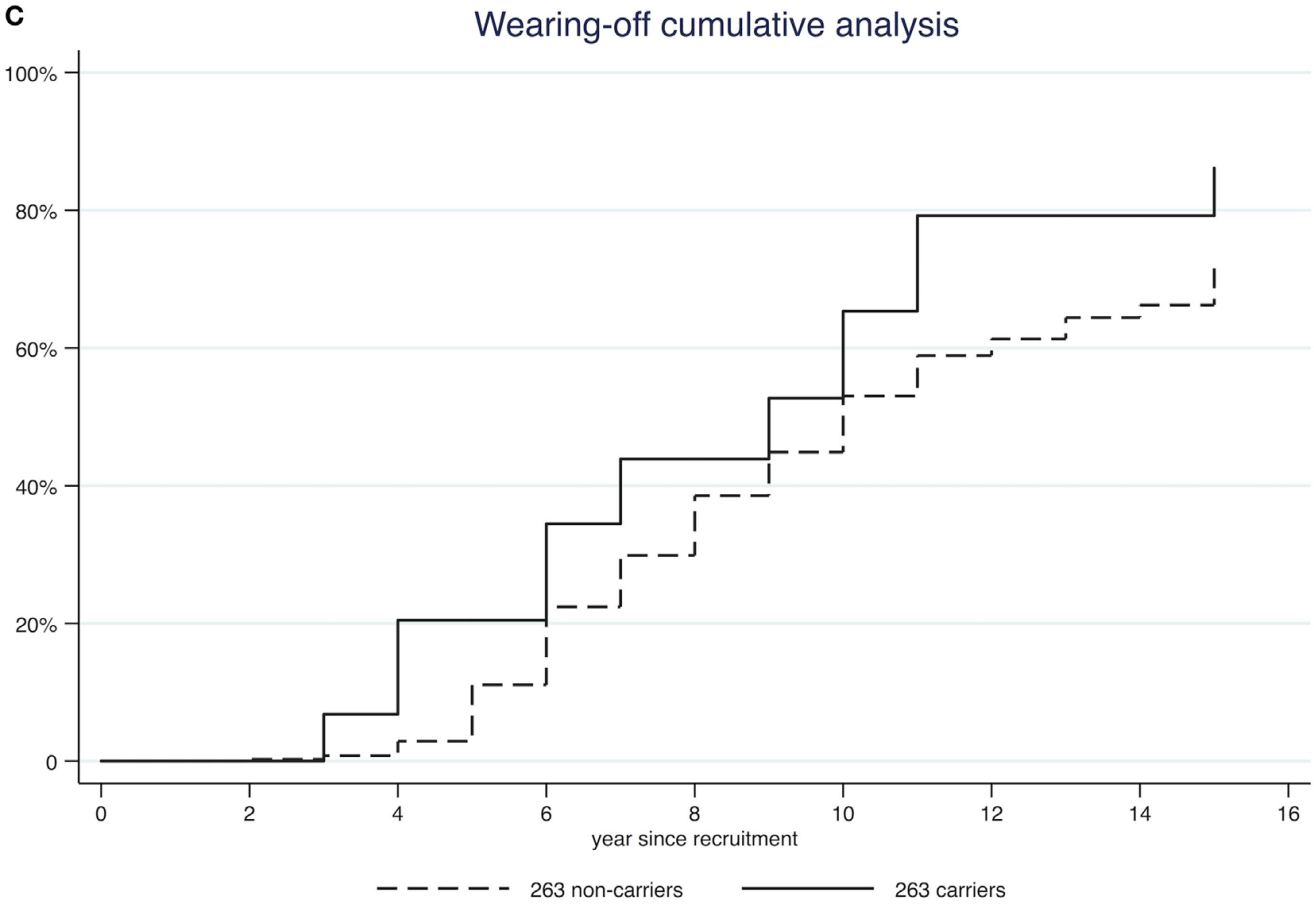
Kaplan–Meyer survival analysis of complications [dementia panel **(A)**; visual hallucinations panel **(B)**; wearing-off panel **(C)**].

Furthermore, we performed a Kaplan–Meyer survival analysis to compare the probability of developing each complication over time in 263 carriers vs 263 non-carriers (Figure 1; Figure S1 in Supplementary Material). At 10 years from onset, cumulative incidence of dementia was higher in carriers than in non-carriers (26.39 vs 12.94%, log-rank test *p* = 0.001; Table 2). Similar findings were also observed for visual hallucinations and wearing-off (24.48 vs 15.01% and 65.33 vs 53.02%, log-rank test *p* = 0.0037 and *p* = 0.028 respectively; Table 2).

**Table 2:** Probability of complications at 10 years from disease onset.

	Rep1 263 carriers % at 10 years (95% CI)	Rep1 263 non-carriers % at 10 years (95% CI)	P*
Wearing-off	65.33 (50.03–80.14)	53.02 (47.47–58.59)	0.028
Dyskinesia	44.10 (29.81–61.55)	44.05 (38.67–49.81)	0.360
Freezing of gait	33.19 (21.07–49.72)	34.25 (29.18–39.92)	0.621
Visual hallucinations	24.48 (13.88–40.97)	15.01 (11.50–19.47)	0.004
Dementia	26.39 (15.50–42.72)	12.94 (9.72–17.13)	0.001

*Log-rank test.

Finally, after applying a multivariate Cox regression model adjusting by gender, age at disease onset, and recruitment center, a statistically significant difference persisted for dementia and visual hallucinations, but not for wearing-off. In detail, we found that 263 carriers had a 3.03-fold higher risk of dementia, a 2.69-fold higher risk of visual hallucinations, and a 1.26-fold higher risk of wearing-off compared to non-carriers (*p* < 0.001, *p* = 0.001, and 0.236, respectively; Table 3).

**Table 3:** Multivariate Cox’s regression model on all study population.

	HR (95%CI)^a^	*P*
Wearing-off	1.26 (0.86–1.86)	0.236
Dyskinesia	0.94 (0.62–1.45)	0.794
Freezing of gait	0.97 (0.61–1.55)	0.901
Visual hallucinations	2.69 (1.57–4.60)	0.001
Dementia	3.03 (1.74–5.30)	<0.001

*^a^Hazard ratio (HR) of Rep1 263 carriers vs 263 non-carriers*.

*Data are adjusted by gender, age at PD onset, and recruitment center*.

## Discussion

This study showed for the first time that Rep1 polymorphic variants of the *SNCA* gene influence non-motor evolution in PD. In patients carrying the *SNCA* Rep1 263 allele, the risk of developing dementia and visual hallucinations is increased by 3.03 and 2.69, respectively, compared to patients carrying shorter Rep1 variants. With regards to motor progression, Rep1 263 carriers display a slightly increased cumulative incidence of wearing-off, compared to non-carriers, even though such difference was not statistically significant in multivariate regression.

To date, there is strong evidence supporting a genetic component in PD susceptibility. On the contrary, few studies have addressed the role of disease modifying genes (19, 20, 21). This might be due, at least in part, to the difficulty in collecting data on disease evolution compared to recording disease development. Consistently, large collaborative studies aimed at tracking PD progression are ongoing (22, 23). In our study, we chose to include only patients with longitudinal follow-up and accurate information regarding the main motor and non-motor complications, putting together a well characterized population of 426 patients.

The cognitive features of 263 carriers recalled that of familial PD caused by rare pathogenic *SNCA* multiplications, in which progression is faster compared to sporadic PD (24). It is known that such mutations cause alpha-synuclein overproduction through increased mRNA expression (25). With regards to the possible effect of the length of Rep1 microsatellite on alpha-synuclein expression, conflicting data were reported. Chiba-Falek et al. detected a threefold increase of alpha-synuclein expression with longer compared to shorter Rep1 alleles in a cellular model (26). Furthermore, alpha-synuclein mRNA varied 1.7-fold in transgenic mice carrying the different Rep1 alleles (27). These findings were further supported by Fuchs et al. who detected a correlation between protein level in blood and Rep1 genotypes (28). Intriguingly, a recent report remarked that plasma alpha-synuclein levels may predict cognitive decline in PD (29). On the contrary, Soldner et al. suggested that neither the deletion of the entire Rep1 repeat sequence nor the presence of the three different length alleles (including 263 bp) were able to influence significantly *SNCA* expression. Nonetheless, the authors themselves underline that their findings might have been influenced by the cellular model analyzed, which only allows to detect early events (30). For such reasons, a robust biologic relationship between the 263 allele and PD progression still has to be confirmed.

Our study is the first to investigate the correlation between *SNCA* polymorphisms and PD progression in the European population. On the contrary, two Northern American studies, the first on 233 patients and the second on 1,098 patients, were published in 2012 and 2014, respectively (12, 13).

In the first study, a faster motor progression in PD patients carrying at least one Rep1 263 was reported (13). As regards, motor progression in Rep1 263 carriers, our findings display a trend similar to that of Ritz et al. (13), even though our data lose statistical significance in the multivariate regression model. Nonetheless, it should be noted that discrepancies might be related to substantial methodological differences. In fact, Ritz et al. (13) studied a cohort of 233 PD patients for a relatively short follow-up (5.1 years) and measured motor progression with the motor section of UPDRS. This score: (a) Does not weigh the different motor signs, (b) does not evaluate cognitive and neuropsychiatric complications, (c) is strongly influenced by treatment, and (d) shows a relevant inter-rater variability (31). On the other hand, our study of Rep1 SNPs included a larger population (426 patients) with a mean follow-up of 11.3 years. Progression was measured through clinical milestones, which are reliable measures of patients’ status, especially when multicenter studies are concerned.

A second report was published in 2014 by Markopoulou et al. (15). In this study, authors failed to detect a detrimental effect of Rep1 263 allele on PD progression. On the contrary, they found that patients carrying longer Rep1 alleles had a better motor outcome and a similar trend was observed for cognitive outcome, even though without statistical significance. In this case too, important differences between the two studies might account for the discordant results. First, our clinical data were collected through direct examination of patients, whereas cognitive data included in the report by Markopoulou et al. (15) were collected through telephonic interviews. Second, the biological effect of Rep1 microsatellite might differ among subjects, especially when a different ethnic background is involved. In fact, Rep1 is a complex microsatellite characterized by a mixed dinucleotide composition. Alleles with the same size could have different composition and it was reported that two alleles with the same length, but different dinucleotide composition might have different functional effects on the promoter activity (26). Finally, the way data are presented in the two studies makes results difficult to compare. In fact, Markopoulou et al. (15) aggregated patients carrying two different genotypes (261–261 and 259–263) in a unique very large group, thus losing the impact of the 263 allele.

## Conclusion

Our study found a significant and biologically plausible correlation between *SNCA* Rep1 263 allele and the risk of development of dementia and visual hallucinations in a cohort of Italian PD patients. Future studies recruiting larger populations and assessing correlations between *in vivo* markers of pathology, such as cerebrospinal fluid, MRI, or PET findings, and genetic variations are mandatory to replicate our findings (32). Moreover, a thorough assessment of further clinical features, such as hyposmia and REM behavior disorder in the genetic subgroups may add relevant information on the determinants of progression (33). Early identification of patients at high risk of complications has relevant implications in terms of both prognosis and disease modifying therapy.

## Ethics Statement

This study was carried out in accordance with the recommendations of the local Ethics Committee with written informed consent from all subjects. All subjects gave written informed consent in accordance with the Declaration of Helsinki. The protocol was approved by the local Ethics Committee.

## Author Contributions

(1) Research project: A. Conception, B. Organization, C. Execution; (2) Statistical analysis: A. Design, B. Execution, C. Review and Critique; and (3) Manuscript: A. Writing of the first draft, B. Review and critique. LC: 1A, 1C, and 3A. FDM: 1B, 1C, and 3A. STu: 2A, 2B. GDO: 1C. MC: 1C. LM: 1C. STe: 1C. GR: 1C. ADF: 1C, 3B. CL: 1C. IT: 1C. RZ: 1C. CCe: 1C. SDA: 3B. CM: 2A. GPC: 3B. GB: 3B. CP: 1B, 3B. RC: 3B. SG: 1B, 3B. CCo: 1A, 1B, 2C, and 3B.

## Conflict of Interest Statement

The authors declare that the research was conducted in the absence of any commercial or financial relationships that could be construed as a potential conflict of interest.

## Supplementary Material

Figure S1 Kaplan–Meyer survival analysis of complications [dyskinesia panel **(B)**; freezing of gait panel **(A)**].

Table S1 Genotype distribution frequencies.

Table S2 Age at PD onset, disease duration and family history for 263 carriers and 263 non-carriers.
