# Supplementary figures and images for: The Length of SNCA Rep1 Microsatellite May Influence Cognitive Evolution in Parkinson’s Disease
Source: Front Neurol. 2018 Mar 29;9:213. doi: 10.3389/fneur.2018.00213 (PMC5890103; doi:10.3389/fneur.2018.00213)

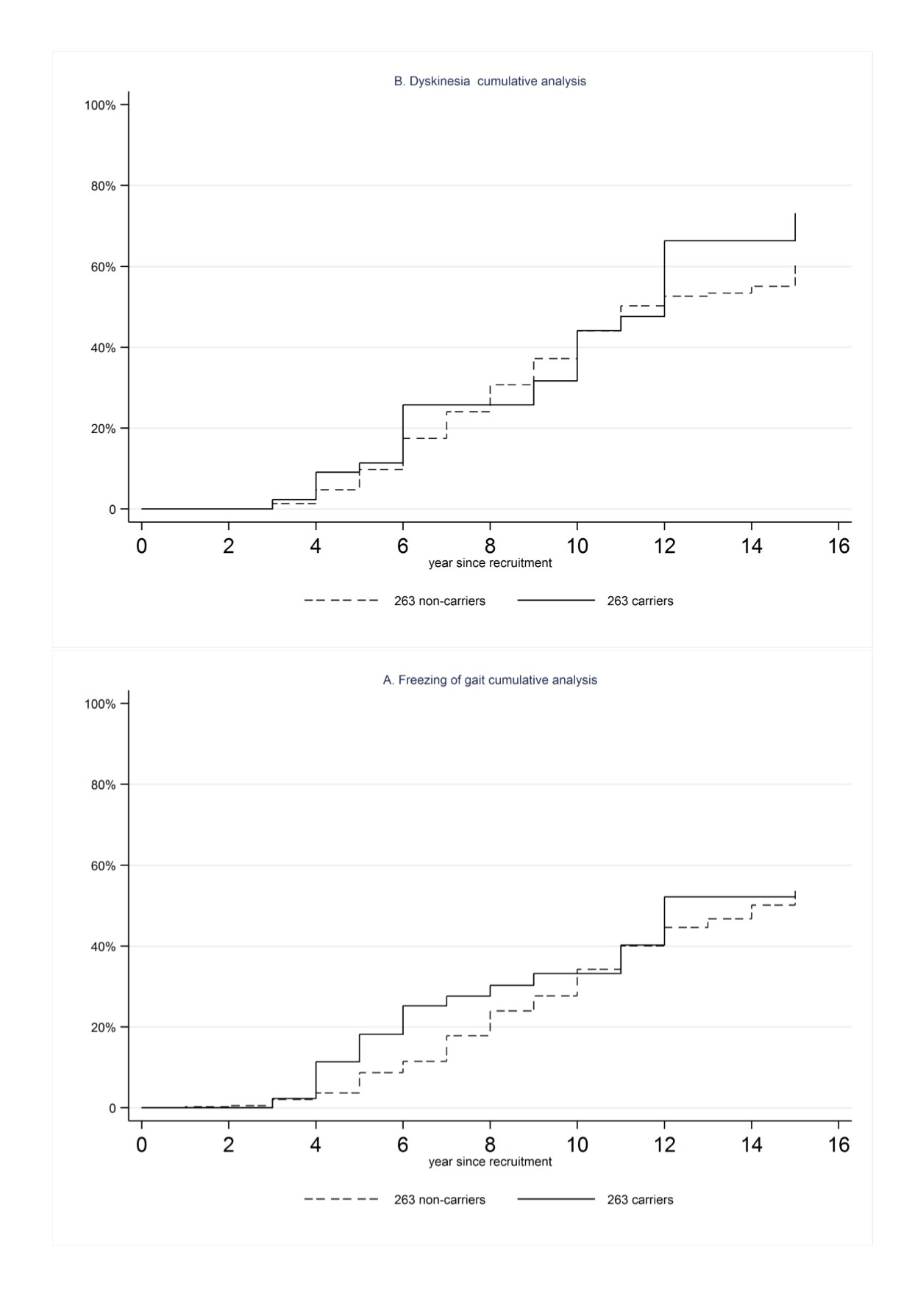

Supplement: Figure S1 — Kaplan–Meyer survival analysis of complications [dyskinesia panel (B); freezing of gait panel (A)]. [file image_1.JPEG]
