# Supplementary material for: The Length of SNCA Rep1 Microsatellite May Influence Cognitive Evolution in Parkinson’s Disease
Source: Front Neurol. 2018 Mar 29;9:213. doi: 10.3389/fneur.2018.00213 (PMC5890103; doi:10.3389/fneur.2018.00213)
Supplement: Table S1 — Genotype distribution frequencies. [file data_sheet_1.DOC]

**Supplementary Table 1. Genotype distribution frequencies.**

|  |  |  |
| --- | --- | --- |
|  |  | **N(%), n=426** |
|  |  |  |
| **Rep 1** | 257/259 | 1 (0.23%) |
|  | 259/259 | 22 (5.16%) |
|  | 259/261 | 147 (34.51%) |
|  | 259/263 | 7 (1.64%) |
|  | 261/261 | 212 (49.77%) |
|  | 261/263 | 35 (8.22%) |
|  | 263/263 | 2 (0.47%) |

**Supplementary Table 2. Age at PD onset, disease duration and family history for 263 carriers and 263 non-carriers.**

|  | **Total population, n=426** | | |
| --- | --- | --- | --- |
|  | 263 carriers, n=44 | 263 non carriers, n=382 | P |
| Age at PD onset  median (25^th^-75^th^) | 56 (53-64) | 62 (55 -68) | 0.016* |
| Disease duration  median (25^th^-75^th^) | 12.5 (9.5-17) | 11 (8-14) | 0.088* |
| Family history, n (%) | 9 (20) | 72 (19) | 0.797^§^ |

*Mann-Whitney U Test

^§^χ2 test
